# Microalgal Biorefinery Concepts’ Developments for Biofuel and Bioproducts: Current Perspective and Bottlenecks

**DOI:** 10.3390/ijms23052623

**Published:** 2022-02-27

**Authors:** Ramachandran Sivaramakrishnan, Subramaniyam Suresh, Simab Kanwal, Govindarajan Ramadoss, Balasubramani Ramprakash, Aran Incharoensakdi

**Affiliations:** 1 Laboratory of Cyanobacterial Biotechnology, Department of Biochemistry, Faculty of Science, Chulalongkorn University, Bangkok 10330, Thailand; rsrkbiol@gmail.com (R.S.); ramksk@gmail.com (B.R.); 2 Department of Biotechnology, College of Science and Humanities, Ramapuram Campus, SRM Institute of Science and Technology, Bharathi Salai, Ramapuram, Chennai 600089, India; sureshbiochem14@gmail.com; 3 Department of Pharmacognosy and Pharmaceutical Botany, Faculty of Pharmaceutical Sciences, Chulalongkorn University, Bangkok 10330, Thailand; simab.k@chula.ac.th; 4 School of Chemical and Biotechnology, SASTRA Deemed University, Thanjavur 613401, India; ramadossgovindarajan1@gmail.com; 5 Academy of Science, Royal Society of Thailand, Bangkok 10300, Thailand

**Keywords:** biofuel, biorefinery, high-value products, microalgae, strain improvement

## Abstract

Microalgae have received much interest as a biofuel feedstock. However, the economic feasibility of biofuel production from microalgae does not satisfy capital investors. Apart from the biofuels, it is necessary to produce high-value co-products from microalgae fraction to satisfy the economic aspects of microalgae biorefinery. In addition, microalgae-based wastewater treatment is considered as an alternative for the conventional wastewater treatment in terms of energy consumption, which is suitable for microalgae biorefinery approaches. The energy consumption of a microalgae wastewater treatment system (0.2 kW/h/m^3^) was reduced 10 times when compared to the conventional wastewater treatment system (to 2 kW/h/m^3^). Microalgae are rich in various biomolecules such as carbohydrates, proteins, lipids, pigments, vitamins, and antioxidants; all these valuable products can be utilized by nutritional, pharmaceutical, and cosmetic industries. There are several bottlenecks associated with microalgae biorefinery. Hence, it is essential to promote the sustainability of microalgal biorefinery with innovative ideas to produce biofuel with high-value products. This review attempted to bring out the trends and promising solutions to realize microalgal production of multiple products at an industrial scale. New perspectives and current challenges are discussed for the development of algal biorefinery concepts.

## 1. Introduction

Microalgae are photosynthetic organisms thriving in aquatic and marine environments with a cell size diameter in the range of 1 to 100 microns. The term ‘microalgae’ is generally used for both cyanobacteria (prokaryotic blue-green bacteria) and unicellular photosynthetic organisms (eukaryotic diatoms). Microalgae contain numerous organic and chemical compounds, which are often considered as secondary metabolites. These metabolites are produced by the different critical metabolic pathways and as intermediates within microalgae. The metabolites can be utilized as a precursor for the synthesis of various important products [1,2]. The advantage of microalgae is that they utilize light energy to grow photoautotrophically with CO_2_ and mixotrophically with sugars. On the other hand, they can grow heterotrophically without light but with organic carbon as a source of carbon and energy [3]. Some microalgae are able to tolerate extreme conditions and grow in diverse environments. The major advantage of microalgae is that they can be cultivated using water and atmospheric carbon dioxide, which can help reduce the production cost compared to other organisms. Utilization of microalgae eliminates the controversies of food vs. fuels. Moreover, microalgae can be grown in degraded land [4]. Microalgae show high photosynthetic efficiencies, which is the important factor for bioenergy production systems using sustainable resources [5].

In addition, microalgae can undergo genetic modifications to trigger the production of various compounds including non-native products. Microalgae such as *Chlorella*, *Dunaliella salina*, *Phaeodactylum*, *Chlamydomonas*, and *Synechocystis* sp. are widely considered for genetic engineering due to the availability of a full sequence database. Recently, various technological improvements have been available for microalgal culturing, harvesting, and extraction. Hence, microalgae are readily available to produce various products such as biofuels, food supplements, cosmetics, aquaculture hatcheries’ feed, pharmaceuticals, lubricants, and other valuable products [6]. So far, microalgae have been extensively studied for biofuel application. Therefore, it is necessary to explore other algae-based, non-fuel products such as omega fatty acids (polyunsaturated fatty acids), proteins, antioxidants, chlorophylls, phycobiliproteins, etc. Among several microalgal sp., only small portions of microalgae have been isolated and identified [7]. Although various known microalgae sp. are available, *Chlorella* and *Spirulina* are the leading sp. used widely for nutrition and other algae-based food products [8]. Major strains, excluding *Chlorella* and Spirulina, have not been used widely for large-scale production due to the lack of studies concerning growth enhancement and the lack of information on high-value products [9]. Researchers and algal technologists continue searching for novel strains in terms of high growth and production of biofuels as well as high-value products. Novel approaches in genetic engineering can help increase the potential of microalgae. New technology such as CRISPR/Cas9 shows highly efficient output in microalgae strain improvements [10]. Currently, more than 15,000 algal genes are fully sequenced and available in GenBank (http://www.ncbi.nlm.nih.gov/gene/ (accessed on 14 January 2022). Future genome studies will help to identify new promising genes, which could be used for algae-based industrial applications.

In this review, the latest trends in biorefinery concepts to produce high-value products were summarized. The fundamentals of microalgae biorefinery and its advantages were evaluated. Various high-value products such as lipids, carbohydrates, proteins, pigments, vitamins, and antioxidants were explored to bring out the potentials of microalgae biorefinery for future applications.

## 2. Biorefinery

### 2.1. Hypothesis

Biorefinery is a process to obtain multiple products from one biomass. Microalgae are mostly considered as feedstocks for the production of biofuels. However, other than the biofuels, microalgae also possess various valuable materials that can be converted or processed into high-value products [11]. Fossil fuel-derived emission is highly responsible for the increase in global warming. The biorefinery concept is considered as a promising solution to mitigate greenhouse gas emissions [12]. Substantial benefits including bioeconomy have been identified in microalgae-based biorefinery. The biorefinery concept also allows for the exploitation of microalgae to their full potential and minimizes biomass waste accumulation. The major bottleneck of microalgae biorefinery is the separation of different fractions into a single, desired fraction. However, this can be overcome by process development in a cost-effective manner utilizing microalgae as the promising candidates to produce high-value compounds in addition to biofuels [13]. Microalgae have advantages in terms of high growth rate, carbon mitigation efficiency, and elimination of the food industry competition with respect to biofuel production [14]. Various microalgae are rich in different valuable compounds. In general, the important biofuel substrates derived from microalgae are lipids and carbohydrates. Other important microalgal fractions such as proteins, minerals, and some alcohols are also used to produce chemicals, feeds, or value-added products. The by-products derived during biofuel formation (for example, glycerol from biodiesel production) can also be considered as value-added products. The residues obtained from thermochemical conversion and other low-value carbohydrate and protein residues are considered for their combined heat and power (CHP) generation. Other than the biofuel, promising by-products should be recovered from microalgal biomass and considered through techno-economic designs, which alleviate the pressure caused by high-cost processing (medium, cultivation, and harvesting) of microalgal biofuel [15].

### 2.2. Microalgal Biorefinery

A recent study reported the success of the microalgal biorefinery approach to produce more than one bioproduct [16]. Two main stages are considered as the important stages in microalgal biorefinery, which are upstream processing and downstream processing. Upstream processing includes the type of microalgae strains, nutrient sources, and light illumination [17]. The nutrient compositions for microalgae cultivation are an important factor for microalgal growth. The variations in nutrient composition improve the various biochemical compositions’ production and microalgal growth. Sivaramakrishnan and Incharoensakdi [18] reported that nitrogen depletion increased the lipid content and decreased the growth rate, whereas the addition of sodium carbonate increased both lipid and biomass content considerably. The study of light intensity effects on microalgal growth revealed that the artificial light source (fluorescent) increased the biomass content more than did direct sunlight due to the high CO_2_ fixation efficiency of an artificial light source [19]. The production of a large biomass and improved high-value products can compensate for the cost of artificial light. Light intensity is the important factor that can highly influence microalgal growth. Hence, it is clear that nutrition and light play a major role in algal growth. 

**Figure 1:**
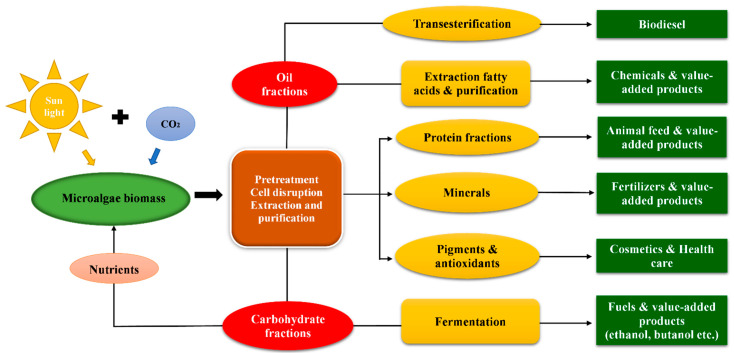
Possible microalgal biorefinery bioproducts.

Another important stage is downstream processing, which includes harvesting, disruption, extraction, and purification of targeted high-value compounds from microalgae. Harvesting is the important process and comprises 20–30% of the total cost of the microalgal biorefinery; it may slightly vary depending on the type of microalgae and technology used. An efficient harvesting technology will reduce the overall cost of microalgal biorefinery at the downstream processing stage. Microalgae harvesting can be done by centrifugation, filtration, flocculation, and floatation or sedimentation processes [20]. Flocculation is considered a highly economical and cheap method when compared to other methods. Flocculation is the process that increases the sedimentation efficiency of microalgae with the help of flocculating agents [13]. Cell disruption is another important downstream process that increases the overall microalgal biorefinery cost incurred by the use of technologies for cell disruption such as homogenizers, bead beating, French press, chemicals, and high-pressure heating [21]. The harvested biomass is further used for biorefinery approaches. The selected methods for biomass conversion should be under mild conditions, without affecting the other fractions [17]. The improvement in technologies involved in downstream processing will have benefits in terms of processing steps and economic aspects. Biofuel is the first option when applying microalgal biorefinery approaches. The major biofuels such as biodiesel, bioethanol, and biohydrogen can be produced by transesterification, thermochemical and biochemical conversions, and photosynthesis-mediated microbial fuel cells [22]. The primary products from microalgae are preferably fuel-based products; the other valuable products are obtained as by-products, which may require other conversion processes. The possible microalgal biorefinery fuels and bioproducts are shown in Figure 1. Carbohydrate, lipid, and protein contents of some important microalgae are listed in Table 1.

### 2.3. Transesterification

Microalgae are considered as a potential feedstock to produce biodiesel. Biodiesel can be efficiently produced by transesterification, in which triglycerides are converted to fatty acid methyl esters and glycerol in the presence of a catalyst and methanol. Ethanol can be used as an alcohol in transesterification. There are several factors that affect the yield of methyl ester such as the quality of oil, nature of catalyst, temperature, selection of alcohol, and impurities present in the oil. The catalysts mostly used for the transesterification are base, acid, and enzyme. Among the catalysts, base catalysts are the most efficient, with the maximum yield achieved in a short duration. However, the presence of free fatty acids in oil during base-catalyzed transesterification leads to soap formation. Hence, acid catalysts are preferred for oil with high free fatty acids’ content. On the other hand, when there are problems during downstream processing of biodiesel production, enzyme catalysts are considered. Although the enzyme catalyst takes a long time to complete the reaction, it can be done under ambient temperatures with ease of recovery of the biodiesel from the reaction mixture. Thermo-stable and solvent-tolerant lipases from *Bacillus* sp. have a high potential for converting triglycerides into biodiesel by transesterification. These enzymes can also withstand high temperatures, which is advantageous to increase the reaction rate. *Scenedesmus* is known for its high-quality lipid, which is used for transesterification [31]. Sivaramakrishnan and Incharoensakdi [32] reported that the enzymatic transesterification of *Botryococcus* sp. can produce biodiesel and the industrially valuable by-product, glycerol carbonate. Methyl ester can be produced by enzyme-catalyzed transesterification from *Botryococcus* sp. followed by the production of bioethanol [16]. As a biorefinery approach, methyl ester was produced from the *Chlamydomonas* sp., and the spent biomass was utilized for the production of ϵ-polylysine [13].

### 2.4. Photosynthetic Microbial Fuel Cells

The conversion of chemical energy to electrical energy through a pair of redox reactions is done using a fuel cell. The cell requires hydrogen as a continuous fuel and oxygen from the air to promote the reaction. Similarly, in microbial fuel cells (MFC) the production of electricity from the biodegradation of organic matter in the absence of oxygen (anaerobic condition) is driven by the bacteria by transferring electrons from the cathode to the anode to maintain the electric current. The recent advancement in these fuel cells (bioelectrochemical devices) shows the greater potential in the production of an oxygen-rich environment, and, at the same time, it removes CO_2_ through the photosynthetic activity of algae, which is integrated with the MFC [33]. The photosynthetic microbial fuel cell is composed of an anode, where the bacteria oxidize the organic compounds and produce electrons, which are then transferred to the cathode through an external circuit to produce electricity. The proton exchange membrane is used to separate the anode and the cathode. The significance of this integrated system involves the presence of microalgae in the cathode, which could carry out CO_2_ fixation simultaneously with the production of bioelectricity [34].

### 2.5. Biochemical Conversion

**Figure 2:**
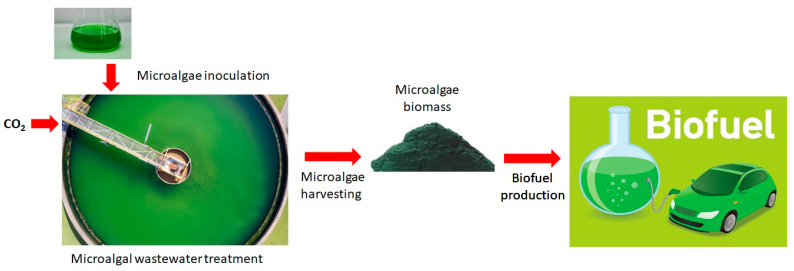
The representation of microalgal wastewater treatment and biofuel production.

The biochemical conversion of biomass into biofuels plays a significant role in energy conversion and production. The biochemical conversion processes include anaerobic digestion for biogas production, alcoholic fermentation for bioethanol production, and photobiological hydrogen production (Figure 2). In the anaerobic digestion process, the conversion of organic compounds and other wastes into CO_2_ and methane is carried out by four processes, i.e., hydrolysis, acidogenesis, acetogenesis, and methanogenesis [35]. The biogas generated from the algal biomass contains a high-energy value, and, at the same time, the energy recovery is on par with the extraction from cell lipids. Due to the depletion of fossil fuels and the increasing cost of energy, the anaerobic digestion of algal biomass is an alternative source for fuel production. In the alcoholic fermentation process, the high carbohydrate content from the cellulose- and hemicellulose (holocellulose)-based cell walls and starch-based cytoplasm is broken down into simple hexose and pentose sugars, followed by the enzymatic fermentation process to obtain bioethanol [36]. Hydrogen from algae is a renewable energy source. The photobiological hydrogen production involves the conversion of water into hydrogen ions and oxygen mediated by algae. At first, the algae are grown photosynthetically and subsequently cultured under anaerobic conditions to stimulate hydrogen production. Then, the simultaneous production of photosynthetic hydrogen and oxygen gas occurs, and then these gases could be separated spatially.

#### 2.5.1. Biogas Production

The use of algae as a potential feedstock for biogas production was addressed in the recent past. The ability of microalgae towards the efficient photosynthetic conversion of sunlight into chemical energy provides the focus on biogas production through anaerobic digestion. It is an economically feasible and friendly process, which converts the entire algal biomass to biogas/methane. Based upon the reported literature, the effectiveness of the fermentation process with various unicellular algae, such as *Melosira* sp., *Oscillatoria* sp. [37], *Spirulina* sp. [38], *Scenedesmus* sp. [39], *Euglena* sp., and *Chlorella vulgaris* [40], or macrophytobenthos organisms, e.g., *Gracilariaceae*, *Laminaria* sp., *Macrocystis* sp., and *Ulva* sp. [41], was used for biogas production. Other species such as *Macrosystis pyrifera*, *Tetraselmis*, *Gracilaria tikvahiae*, *Hypnea* sp., and *Ulva* sp. may prove efficient as organic substrates in methane fermentation processes. Algae biomass can be effectively utilized for biogas production from natural, eutrophicated, and degraded waterbodies.

The utilization of algal biomass from the natural environment created a massive production of biomass, approximately 100 tons a day, which serves as a potential source of organic matter for the production of biogas [42]. The algae biomass is grown under controlled conditions mainly with open and closed installation designs. These designs eradicate the bottlenecks observed during algae cultivation in natural water bodies. Concrete ponds, circular ponds with agitators, and raceway ponds with paddle and cascade ponds are considered as open installation designs. The closed design using photobioreactors provides constant control of various operating parameters, such as intensity of light, time of exposure to light, and temperature of the growing medium, and decrease the possibility of the risk of parasites and other organisms [43].

Golueke et al. [44] reported the first attempt using a mixed culture of *Chlorella* sp. and *Scenedesmus* sp. for methane fermentation. In addition, the effectiveness of the fermentation process was compared between algae biomass and sewage sludge, which showed 1020 dm^3^/kg organic dry matter (o.d.m) for the sewage sludge and 986 dm^3^/kg o.d.m. for the algae biomass [45]. From the result, it was concluded that the production of biomass per kg of organic dry matter and the qualitative analysis of gaseous metabolites were comparable for both substrates. The concentration of methane in biogas ranged from 61 to 63%. 

Klassen et al. [46] reported that cultivation of microalgae under nitrogen repletion and limited conditions led to the formation of protein-rich and low-protein biomasses, respectively. Anaerobic digestion of nitrogen-limited biomass resulted in high biogas and methane productivity of 750 and 462 mLN g^−1^ volatile solids (VS) day−1, respectively. The corresponding energy conversion efficiency of biomass to methane was found to be 84%. Based on these results, the highly efficient anaerobic digester revealed a clear predominance of the phyla *Bacteroidetes* and the family *Methanosaetaceae* among the Bacteria and Archaea, respectively. Depending upon the microalgal species and co-digestion system, the biogas/methane yield was improved by 4–260% compared to a mono-digestion process [47]. The biogas yield from co-digestion may also vary depending on the type of temperature conditions (thermophilic or mesophilic) of anaerobic digestion. According to a recent study, no significant difference was observed in the average methane yield between the thermophilic co-digestion of 12 species (318 N cm^3^ g^−1^ VS) and mesophilic co-digestion of 29 species (317 N cm^3^ g^−1^ VS) [48].

Pretreatment of the biomass, co-digestion of microalgae, strain improvement, and an integrated biorefinery approach are the strategies to be implemented for the enhanced production of biogas and sustainability. Algal biomass represents a potential green bioenergy source for the production of biogas through anaerobic digestion. However, the algal biogas production process is currently unsuccessful, unprofitable, and unsustainable unless there is an implementation of a circular economy such as wastewater treatment, reduction in eutrophication, or zero-waste objectives in algal biorefineries.

#### 2.5.2. Bioethanol Production

The rise in the global population and the current development of highly populated countries such as India and China have contributed to an increase in energy demand. Fossil fuels such as oil and natural gas cannot meet the current demand of consumption. The annual increase in worldwide energy consumption in the last two decades has triggered the interest in finding alternative energy sources such as biofuels. The long-term technological development of biofuels has been classified into four generations [27]. First- and second-generation biofuels are produced from cellulosic biomass, non-food crops, agricultural wastes, and energy crops. Biofuels produced from algae are third-generation biofuels, commonly known as algae biofuel. The biofuels produced from most advanced and genetically engineered microbial systems are termed as fourth-generation biofuels. Bioethanol is a biodegradable and eco-friendly fuel produced from various feedstocks such as cellulosic biomass, agricultural and other lignocellulosic wastes, etc. Bioethanol is produced from carbohydrates such as simple sugars, which are fermented to produce bioethanol and carbon dioxide, as shown in Equation (1):

C_6_H_12_O_6_ → 2C_2_H_5_OH + 2CO_2_ + heat (1)

Lignocellulosic biomass (LCB) is cheap and plentiful; however, converting the biomass into ethanol is more expensive compared with other feedstocks. Similar to LCB, algae also undergo pretreatment, hydrolysis, and fermentation to produce bioethanol. Cell walls and starch-based cytoplasm contain a higher composition of carbohydrate, which can serve as a suitable feedstock for bioethanol production. During enzymatic hydrolysis, these carbohydrates are hydrolyzed into monomeric sugars, which, in turn, are converted into bioethanol by fermentation. 

Starch-rich microalgae have been studied for the production of bioethanol. Different pretreatment methods have been employed to release the fermentable sugars in order to enhance bioethanol production. Microalga genera such as (1) *Gracilariaceae*, (2) *Dunaliella*, (3) *Chlorella,* (4) *Chlamydomonas,* (5) *Nannochloropsis*, (6) *Scenedesmus*, (7) *Oscillatoria, and* (9) Spirulina are some of the most abundant algae existing in salt or fresh water. There are several advantages when utilizing algae as a feedstock for the production of biofuel, such as a short harvesting time, cheap farming, mitigation of CO_2_, high-efficiency process, faster growth rate of biomass, and less consumption of water compared to conventional crop feedstocks. 

The fermentation of algal biomass to bioethanol comprises four different steps: (1) pretreatment, (2) hydrolysis or saccharification, (3) fermentation, and (4) product recovery. Algal biomass is an attractive feedstock because, unlike other lignocellulosic biomass such as rice straw, wheat straw, or bagasse, algae contain no lignin and, hence, the lignin removal step is not necessary. Lignin removal is a rate-limiting step for other feedstocks; hence, the use of algae reduces costs, time, and conversion process [49]. The efficiency of each step would influence the final ethanol yield; therefore, each process condition is carefully selected and optimized to maximize the product yield. One of the most important steps in bioethanol production from algal biomass is pretreatment. This process is used to make the biomass more susceptible to further breakdown by separating the cellulose and hemicellulose fractions. All the pretreatment methods have intrinsic advantages and disadvantages. The formation of inhibitors, high-energy requirements, catalyst requirements, degradation of sugars, and catalyst recovery are some of the disadvantages of pretreatment. 

The hydrolysis of algal biomass (particularly macroalgae) involves the cleavage of polymeric units of compounds such as alginate, agar, cellulose, carrageenan, laminarin, mannitol, and ulvan. The simple monosaccharides’ sugars recovered from algal biomass include glucose, galactose, rhamnose, mannose, fucose, xylose, and arabinose for the production of bioethanol through the fermentation process. The most commonly used methods for the hydrolysis of algal biomass are dilute acid, alkaline, enzymatic, and thermal hydrolysis. In general, enzymatic hydrolysis has been promoted because the enzymes are considered more eco-friendly in their application and they generate no inhibitors during the process. 

As with other feedstocks, bioethanol from algal biomass can be produced by four different processes: (1) separate hydrolysis and fermentation (SHF), (2) simultaneous saccharification and fermentation (SSF), (3) simultaneous saccharification and co-fermentation (SSCF), and (4) consolidated biomass processing (CBP). Among the various processes, SHF is the most common and well-developed approach that allows the use of the optimal conditions for both the hydrolysis and fermentation processes. Various microalgae can produce ethanol by using different pretreatment and fermentative organisms. Some important microalgae are summarized in Table 2.

#### 2.5.3. Biohydrogen Production

Hydrogen from microalgae is considered as the alternative to replace fossil fuel-based energy requirements; it is clean and non-toxic. Hydrogen has a high-calorific value of ~122 kJ/g compared to other fuels; it has a 2.75-fold higher heating efficiency compared to other hydrocarbon fuels [58]. In addition, during combustion, hydrogen (H_2_) releases water (H_2_O) as its by-products. H_2_ is a renewable fuel and it is a clean energy, which can be obtained naturally from the Earth. H_2_ can be produced from waste biomass, steam reforming, syngas utilization, coal, and natural gas [59]. The conventional methods for H_2_ production are highly energy intensive and can be done at very high temperatures ranging from 970—1100 K. This energy-intensive process also produces carbon dioxide, which is not economically feasible for commercial scale [60]. Biological hydrogen production can be produced by cyanobacteria, microalgae, and photosynthetic microorganisms through microbial biophotolysis and fermentation. Biophotolysis can be done by utilizing environmental H_2_O and direct sunlight. With the help of sunlight, H_2_O splits into H_2_ and O in the bioreactor atmosphere, and H_2_ can be collected and utilized for the energy generation [61]. When compared to thermochemical processes, biological photolysis is eco-friendly and economical. Different organic sources such as lignocellulosic materials, industrial waste, wastewater sludge, rice mill wastewater, and household waste can serve as a substrate for hydrogen production, owing to their starch, lipid, and protein contents [62]. However, the major drawback in biological hydrogen production is oxygen generation. Most of the hydrogen production enzymes are oxygen sensitive and lead to a low hydrogen production yield. Special operating systems and conditions are required to achieve a high production yield [63]. Novel technologies such as genetic engineering are needed to make oxygen-insensitive enzymes for biohydrogen production.

### 2.6. Thermochemical Conversion

The principle of thermal decomposition of organic substances present in the biomass to produce the fuel products is termed thermochemical conversion, which includes direct combustion, pyrolysis, gasification, and thermal liquefaction processes. All these four processes basically require a higher temperature to convert substrates into useful products. As the name indicates, the direct combustion process involves the reaction between the fuel and oxygen, where this process generates CO_2_, water, heat, and ash as products [64]. Through this biomass combustion process, a high amount of energy is produced; increased efficiency could be attained with a co-combustion process in coal-fired power plants. However, the pyrolysis process is the method where the thermal degradation occurs without the presence of oxygen (anaerobic process), and it is mostly employed in large-scale production, which produces fuels with low-calorific power [65]. Gasification is the process of converting carbonaceous materials into synthesis gas (syngas). The syngas has the potential to burn directly as a fuel for gas engines or it can be used to manufacture wide varieties of chemical intermediates. The last process in the thermochemical conversion is thermal liquefaction, where the algal biomass undergoes liquefaction and is decomposed into molecules with high energy density. 

### 2.7. Bioplastics

Many microorganisms can produce polyhydroxybutyrate (PHB), which can be stored as an energy material. PHB thermoplastic obtained from bacteria is 100% biodegradable with a similar characteristic to polypropylene [66]. However, commercial PHB production is still not achieved due to the requirement of an expensive sugar substrate and uninterrupted oxygen supply. PHB can also be produced by cyanobacteria and microalgae with the same quality as that obtained from bacteria. Important cyanobacteria that produce PHB are Synechococcus sp., Nostoc muscorum, and Calothrix scytonemicola TISTR 8095 [67]. A photosynthetic organism is widely used for PHB production due to its high proliferating capacity without competing with food crops and without the requirement of an expensive sugar substrate [68]. Cyanobacteria are the primary choice for PHB production. This is due to their ability to grow in wastewater and remove excess phosphate and nitrogen along with PHB production [69]. Media optimization such as phosphate and nitrogen limitation can enhance the PHB production in Synechococcus sp. [70]. There has been a report that phosphorus and nitrogen starvation improved PHB production 9.5%, and the addition of 0.1% glucose and 0.4% acetate produced 29% (*w*/*w*) of PHB in *Synechocystis* sp. PCC 6803 [71]. Isolated *Chlorella* pyrenoidosa showed 27% of PHB content after 14 days of growth [72]. Another study reported that the *Chlamydomonas reinhardtii triglycerol* was molded as bioplastic beads that could withstand 1.7 MPa of compressive stress [73]. Acetate and citrate supplementation in the media is another strategy to improve the PHB production in cyanobacteria and microalgal cells [74]. Strain improvement such as mutation and genetic modification of key enzymes involved in the PHB synthesis pathway can enhance PHB production [75]. Novel photobioreactors such as a bubble column or stirred tank reactor were helpful in the enhancement PHB production [76]. Nevertheless, the technologies are not sufficient to produce the PHB at an industrial scale. Hence, the optimization of a growth medium, genetic modification, and suitable bioreactors can enhance the possibility of PHB production at a large scale.

### 2.8. Potentials of Microalgae for Biorefinery

Microalgae have the potential to produce biofuels, food, value-added compounds, and compounds for cosmetics, chemical industries, and feed [77]. Apart from the fuel applications, microalgae can produce various health-benefitting nutrients such as proteins, essential amino acids, omega fatty acids, vitamins, minerals, dietary fiber, and antioxidants [78]. In general, microalgae reserve a large number of biopolymers, which can serve as an emulsifier or stabilizer and texturizer. In addition, microalgal biopolymers can be used in food hydrocolloids [79]. The major carbohydrate composition in microalgae is starch or cellulose with no lignin content. Hence, microalgal carbohydrates can be easily transformed into biobutanol or bioethanol by fermentation [80]. Various microalgae produce polyunsaturated fatty acids such DHA and EPA, which are omega fatty acids highly beneficial to health [81]. 

## 3. Valuable Biochemical Compositions Available in Microalgae

### 3.1. Lipids

The percentage of lipids in microalgae is around 15 to 80% of their total weight. The lipid content may vary according to the culture conditions, availability of the C/N (carbon to nitrogen) ratio in the medium, and induction of stress conditions. During nitrogen limitation conditions, the microalgal cell embeds more lipids than that under the condition with nitrogen. To enhance the lipid productivity, various strategies to induce stress have been considered such as high temperature, nitrogen starvation, nutrient composition modifications, salt concentration, and altering the pH [82]. Proper stress conditions or other engineering strategies can increase lipid productivity in microalgae. This can be a suitable alternative source for biodiesel production when compared to other oil-based crops, in addition to its becoming a food supplement [83]. Microalgal lipids can be extracted by using different methods such as solvent extraction, microwave-assisted extractions, electroporation, and ultrasonic extraction. However, the major setback of the lipid extraction is that it is energy intensive, requires an enormous number of solvents depending on the nature of the lipids present in the microalgae, and has high operating temperatures and high flammability risks [84]. 

Supercritical extraction methods were recently considered for the replacement of toxic solvents; the major advantage of this method is that it shows high selectivity towards triglyceride, which is the important compound required for biodiesel production. CO_2_ is widely considered for the supercritical extraction of lipids; it is considered safe, economic and environment friendly, and recyclable [84]. However, the major drawback of a supercritical method is that it requires high energy, which affects the overall process. Novel methods have evolved in lipid extraction without using solvents, such as isotonic extraction, osmotic pressure extraction, and enzyme-based extraction. Compared to solvent methods, solvent-free methods are considered as environmentally friendly due to the absence of a solvent; they are also easy and simple. The oil-extracted biomass (spent biomass) can be further utilized for other purposes such as animal feed or ethanol production [85]. 

### 3.2. PUFA (Polyunsaturated Fatty Acids)

The important, essential nutrient required to prevent cardiac disorders is PUFA (polyunsaturated fatty acids), especially omega fatty acids such as EPA and DHA. Primary sources for omega fatty acids are from marine fish. However, due to the pollution of marine environments, the demand for PUFA has been increased day by day; hence, it is necessary to develop new resources for future PUFA demand. Microalgal PUFA is considered as the alternative for PUFA from marine fish; it is much needed for human nutrition and health [86]. Novel technologies can be implemented to produce a high amount of PUFA from microalgae for sustainable production. The EPA production was also enhanced by the type of photobioreactor with a light source arrangement; for example, LED lights of blue or red illumination can induce EPA production [87]. For the extraction of PUFA from microalgae, supercritical fluid extraction was considered as the effective extraction method for DHA and EPA [88]. Different plant hormone treatments enhance the lipid content. For example, the plant hormone indole acetic acid (IAA) enhances the growth of omega fatty acids considerably in *Chlorella* sp. This treatment upregulates the desaturase enzymes and also creates oxidative stress in the *Chlorella* sp., enabling the high biomass and omega fatty acid contents [89]. Another study reported that the transesterification of omega fatty acids-rich *Aurantiochytrium* sp. KRS 101 produced ethyl esters, which can be directly utilized for the treatment of hypertriglyceridemia [90]. Hence, PUFA from microalgae is considered as the important high-value compound for the future PUFA demand.

### 3.3. Carbohydrates

In general, microalgae accumulate about 50% of carbohydrate due to the photoconversion ability of the microalgae; they store a majority of the energy as carbohydrates [91]. The major carbohydrate constituents of microalgae are starch, cellulose, glucose, and other pentoses or polysaccharides. For biofuel production, microalgae rich in cellulose and starch or glucose are widely considered. On the other hand, polysaccharides in the microalgae have various biological functions such as protection, a storage component, and structural maintenance of microalgae [92]. Recent studies have shown that polysaccharides from microalgae can be used as biologically active molecules to boost the immune system or inflammatory reactions. Biologically active carbohydrates include food ingredients, natural therapeutic agents, and cosmetic additives. In general, carbohydrates are extracted from the hydrolysis of microalgae by cleaving long chain polysaccharides into monomers. The widely preferred hydrolysis method was chemical pretreatment or physical pretreatment to achieve fermentable sugars for fermentation [93].

### 3.4. Proteins

Protein is considered to be an important major component for microalgal biorefinery due to its precious nutritive benefits or animal feed. The microalgal cell wall is composed of hemicellulose, cellulose, polysaccharides, and β-glucans. It is necessary to choose the suitable cell disruption method for different microalgae according to their cell compositions [94]. In general, mechanical cell disruption, such as high-pressure homogenizers or bead mills, and non-mechanical disruption using enzymes, chemical agents (e.g., sodium hydroxide), or physico-chemical methods are used to disrupt the cells [95]. After the disruption, microalgal proteins are separated based on the water dispersibility from the cell debris and solid phase [96]. The concentration of microalgal proteins is the next step in protein separation. Specific proteins should be separated from the protein mixture. It can be done through precipitation by using ammonium sulfate or protein-precipitating chemicals [97]. The concentration of proteins can also be done by ultrafiltration methods using semipermeable membranes [98]. Freeze-drying and spray-drying techniques may also be used for protein concentration [99]. The protein is then fractionated according to its ionic property by altering the pH and gradient centrifugation techniques [100]. To achieve a commercial value of the proteins, it is necessary to purify the protein, which can be done using molecular exclusion, ion exchange, affinity, and hydrophobic interaction chromatography [100]. However, it is necessary to identify the suitable proteins because some proteins may be toxic. Generally, proteins are isolated by solvent extraction from the microalgae or microalgal culture. Other harvesting methods such as centrifugation and filtration may lead to the loss of proteins. Hence, solvent extraction is widely preferred to achieve the maximum yield of a protein. It is necessary to maintain a suitable solvent, which should ensure the suitable pH, type of salts used, and ionic strength [17]. Bjornsson et al. [101] attempted a supercritical carbon dioxide protein extraction method, which showed a high protein yield. The supercritical extraction method eliminates the use of toxic and flammable solvents, which is an added advantage. Buchmann and Mathys [102] reported that the microalgal proteins can be extracted efficiently by using a pulsed electric field, which could be much more suitable for the biorefinery concepts. The ϵ-polylysine is a high-value protein; it can be produced by Streptomyces’ fermentation of sugars extracted from *Chlamydomonas* sp. [13].

### 3.5. Pigments 

The important pigments generally found in microalgae are chlorophyll, carotenoids such as xanthophylls (astaxanthin and lutein) and carotenes (α-, β-, and γ-carotene and lycopene), and phycobilin. These pigments have vital roles in various types of nutrition. They could be a vitamin precursor in food or animal feed. The microalgae pigments’ applications are diverse such as for additives, cosmetics, biomaterials, food coloring agents, and pharmaceutical industries [103]. Chlorophyll is the important photosynthetic lipid-soluble pigment; it plays a vital role in photophosphorylation. It can be extracted from microalgae by using traditional solvent extraction methods. Due to the low polarity of the chlorophyll, it is necessary to select a suitable solvent for the extraction. The important factors influencing the chlorophyll content are the cell disruption type, solvent selection, and extraction time. Among the various solvents, methanol and ethanol showed high efficiency towards chlorophyll extraction [104]. For the pretreatment methods, ultrasound cell disruption was found to be very efficient for the extraction of chlorophyll from *Chlorella* sp., *Dunaliella* sp., and *Nannochloropsis* sp. [105]. Chlorophyll can also be efficiently extracted by a supercritical fluid method. However, the major drawback of the supercritical fluid extraction is the requirement of higher temperatures and pressure, which make the process expensive.

Carotenoid plays an important role in coloring agents; it is a natural coloring pigment found in plants and it is a fat-soluble pigment. The important role of carotenoid in plants and microalgae is to absorb the light, which is further used in photosynthesis and limits the photon damage of chlorophyll [106]. Microalgal carotenoid can be extracted by a solvent of Soxhlet extraction methods. The selection of a solvent is most important to extract all the carotenoid content; a non-affinity solvent decreases the final yield and requires more solvent. Carotenoid can also be extracted by using supercritical fluid extraction; the addition of a co-solvent with a supercritical fluid enhances the carotenoid extraction efficiency [106]. Cell disruption is the important step to extract the pigments from the cells. A study reported that the extraction of carotenoid and zeaxanthin was done by different disruption methods. The cell was disrupted by chemical methods such as by using dimethyl sulfoxide (DMSO), hydrochloric and sulfuric acids, and citric and acetic acids; it was found that strong acids such as hydrochloric and sulfuric acids showed a low yield. On the other hand, mild acids such as citric and acetic acids showed high pigment extraction efficiency [107]. Ultrasound cell disruption is the primary choice in the mechanical cell disruption for the pigment extraction; it could be 40 times more efficient when compared to the direct pigment extraction. Ultrasound extraction not only extract the pigments but also disperses the large molecules into small molecules, which helps in achieving a high yield [108]. Both chemical and mechanical disruptions showed significant improvement in a high carotenoid yield compared to direct extraction [109]. 

Another important microalgal pigment is phycobilin, which acts as a photosynthetic accessory pigment; it has numerous applications such as antioxidant, anticancer, antiviral, anti-inflammatory, anti-allergic, as a neuroprotective agent, and as a coloring agent in the food industry and pharmaceutical applications. Phycobiliproteins are light-harvesting protein complexes comprising three different types such as allophycocyanin, C-phycocyanin, and phycoerythrin [2]. Phycobiliproteins have high fluorescent activity; hence, they are used for reagent labeling in flow cytometry, immunohistochemistry, fluorescence immunoassay, and other biomedical sciences. The microalga Spirulina platensis has a high amount of phycobiliproteins in the form of C-phycocyanin, which is a natural blue pigment protein [110]. Phycobiliproteins can be extracted through consecutive repetitions of a freeze-and-thaw process, drying, homogenization, and centrifugation. However, extraction of phycobiliproteins is a time-consuming process and involves numerous steps with the recovery of a low yield [111]. Porav et al. [112] purified the cyanobacterial phycobiliproteins efficiently, with more than 80% of phycobiliproteins (phycocyanin and allophycocyanin) achieved by using a sequential aqueous two-phase system.

Astaxanthin is a microalgal pigment that has various applications; it has a higher antioxidant property when compared to the carotenoid. It also has strong sun-proofing, anti-inflammatory, and anti-aging properties, which were widely used in the cosmetic industries. Astaxanthin can boost the immune system and its applications extended towards the feed, nutraceutical, and food industries [113]. Astaxanthin is oxygen, light, and heat sensitive and is damaged easily when exposed to solvents during solvent extraction and oxidative stress. However, supercritical extraction of astaxanthin shows a high efficiency due to the high solubility of astaxanthin in a supercritical carbon dioxide extraction mixture. In addition, using suitable cell disruption techniques can lead to a high astaxanthin recovery during an extraction process [114]. The important bioproducts’ purification and their yield are presented in Table 2.

### 3.6. Microelements 

Microalgae have high levels of trace elements and vitamins, which strike the top of the table when compared to other commodity feeds. The biological studies on this compound are limited, and the quality of the feed should be ensured. Ljubic et al. [115] reported that the *Nannochloropsis oceanica* can produce 1 µg/g dry cell weight of vitamin D3, and the production was significantly enhanced by UVB stress conditions. The produced vitamin D3 can be used as an animal feed or other direct sources [115]. Another study reported that the cyanobacterium Anabaena cylindrica has a high amount of vitamin K1 (200 µg/g dry cell weight), which is six times higher than other rich dietary sources such as parsley and spinach. One gram of dry biomass can provide three times that of a single adult’s intake per day of vitamin K1; this was increased to 4-fold after optimizing the growth condition. In addition, analysis of the same organism showed that it was rich in vitamin B12, protein, and phylloquinone. An animal study was performed to determine the toxicity of microalgae, and no acute toxicity was found [116]. The nitrogen concentration of the microalgal medium influences the vitamin concentration of the cells. Low nitrogen decreased the vitamin content, whereas a sufficient amount of nitrogen produced a high amount of vitamin (Bonnet et al. 2010). Edelmann et al. [117] reported that *Chlorella* sp. and *Nannochloropsis gaditana* contain 21 to 41 µg/g dry cell weight of riboflavin and 0.13 to 0.28 mg/g dry cell weight of niacin, respectively. In addition, *Chlorella* sp. showed a higher folate content than that of Spirulina and N. gaditana sp.; a predominant amount of vitamin B12 was also found in these organisms. Some microalgal bioproducts and their yields are shown in Table 3.

## 4. Clinically Important Compounds

### 4.1. Anticancer Agents

Microalgae are the promising resources to produce bioactive chemicals that have various health benefits in which an anticancer property is of particular importance. The important microalgal pigments such as chlorophylls, carotenoids, and pigment-associated compounds have been effective for treating cancer cells [2]. Astaxanthin is the important pigment found in microalgae and cyanobacteria, which is also considered for an anticancer drug [124]. Phycobiliproteins from Spirulina are the important compounds that act as important free-radical scavengers and can serve as an antitumor or anticancer drug [125]. Phycobiliprotein, considered as the major accessory pigment in microalgae, has high anticancer, anti-allergic, and anti-inflammatory properties [126]. *Dunaliella salina*, a halotolerant alga that is rich in β-carotene isomers, can serve as an anticancer agent and antioxidative agent. *Dunaliella salina* contains carotenoid derivatives such as β-carotene, α-carotene, zeaxanthin, lutein, and cryptoxanthin [127]. Phycocyanin is a water-soluble pigment found in Spirulina; about 6–7% of its total weight is known for anticancer drugs [128]. A cytotoxicity assay was carried out to determine the effectiveness of microalgal pigments and the results showed that the anticancer activity under nitrogen deprivation conditions was relatively high when compared to control [129]. The cyanobacterium *Scytonemapseudo hofmanni* synthesized some anticancer compounds such as toyocamycin, scytophycin B, and tubercidin, which are highly effective against cancer cells [130]. Another important compound, cryptophycin, a metabolite produced by *Nostoc* ATCC 53789, is considered as an effective component against cancer cells [131]. Macrolides called acutiphycins produced from *Oscillatoria acutissima* showed anti-cancer activity against lung carcinoma [132]. In addition to the pigments, some other components present in the microalgae can also show anticancer activities. Marine diatoms *Thalassiosira rotula*, *Skeletonema costatum,* and *Pseudo-nitzschia delicatissima* produced polyunsaturated aldehydes with anti-proliferative activity on cell lines of human colon adenocarcinoma (Caco-2) [133]. Another study reported that polysaccharides isolated from the *Synedra acus* (diatom) showed anti-tumor activity on DLD-1 cell lines (human colon cancer cell lines) [134]. Violaxanthin is another algal compound extracted from the *Dunaliella tertiolecta* with strong anticancer activity against MCF-7 cancer cells [135]. Eicosapentaenoic acid (EPA), the omega fatty acid present in several microalgae, has high anticancer activity. EPA from *Cocconeis scutellum* (marine diatom) has high potency against BT20 and MB-MDA 468 human breast cancer cell lines and LNCaP human prostate adenocarcinoma cell lines [136]. Kim et al. [137] reported that the stigmasterol from *Navicula* incerta showed efficient anticancer activity against HepG2 human liver cancer cell lines. The stigmasterol can also induce apoptosis [138]. Nanoyl-8-acetoxy-6-methyloctanoate (fatty alcohol ester) and monogalactosyl glycerols from the *Phaeodactylum tricornutum* showed efficient anticancer activity against the HL-60 mouse epithelial cell lines [139,140]. Fucoxanthin from microalgal extracts showed strong anti-proliferative activity against HL-60 cells. In addition, fucoxanthin was used to treat the Caco-2, DLD-1, and HT-29 colon cancer cell lines [141]. It is clear from the previous reports that the natural pigments and some high-value components available in microalgae are considered as potential anticancer agents. However, the designing of an anticancer drug requires more research before it can be applied as a commercial drug.

### 4.2. Antiviral Compounds

Several studies have reported that cyanobacteria and microalgae efficiently produce antiviral products. The compounds from the microalga *Cochlodinium polykrikoides* showed antiviral activity against HSV-1 (herpes simplex virus-1) and influenza viruses [142]. The microalga *Chlorellaceae* and cyanobacterium *Leptolyngbya* sp. contained seven active compounds that inhibited the seasonal influenza viruses A and B in MDCK (Madin–Darby Canine Kidney)-infected cells [143]. The sulfated polysaccharide fractions from red algae *Kappaphycus alvarezii*, *Porphyridium*, and *Hypnea musciformis* showed strong protective activity against HIV-1-induced T-lymphoblastic cells [144]. A ZK antigenic protein served as a vaccine against the Zika virus; it can be produced from *Schizochytrium* sp. by expressing viral vector proteins through algevir technology [145]. A recent study revealed that the *Porphyridium* sp. exopolysaccharides and sulfated polysaccharides could effectively defend against COVID-19 (SARS-CoV-2) [146,147]. According to the FDA (Food and Drug Administration), algal polysaccharides are the acceptable compounds for human and animals. However, the commercial production of antiviral agents is still unexploited. Investigation focusing on microalgal compounds can bring about the discovery of new chemicals that have strong antiviral activities.

### 4.3. Anti-Inflammatory Products

During pathogenic bacteria or virus infection, physical wounds activate complex physiological process called inflammation. It is a common cause to human beings, who can be treated by some microalgal components such as microalgal pigments and PUFAs. The components were considered as potential elements and can be used in diet to alleviate the effects of chronic inflammatory diseases [148]. Astaxanthin from *Haematococcus pluvialis* showed anti-inflammatory activity against inflammation caused by UV radiation, which also stimulates the production of immunoglobulins A, M, and G and T-helper cell antibody in human peripheral blood mononuclear cells [149]. The cyanobacterium *Stigonema* synthesized a protein called scytonemin, which is known for anti-inflammatory action against inflammation and also acts as a serine/threonine kinase inhibitor [150]. *Spirulina* showed high anti-inflammatory activity in toxicity-induced rats and anti-inflammatory action against hematologic, hepatic, and renal biomarkers [151]. Deng and Chow [152] studied the effect of inflammation by using microalgal anti-inflammatory products under in vivo conditions. The results showed that the components such as carotenoids and some pigments from microalgal extracts efficiently reduce the inflammation, and those components showed antioxidant activities.

## 5. Ecological Valuable Compounds

The pesticides used in agriculture are mostly synthetic and strong chemicals, which create several ecological problems. The residues and harmful chemicals present in agricultural crops are toxic and harmful to animals, wildlife, and humans. Those chemicals are the causes for neurotoxicity, endocrine disruption, Parkinson disease, cancers, and type 2 diabetes [153]. The study also discussed and provided evidence about the side effects of various pesticides on humans [153]. Using pesticides indirectly affects the ground water quality, and the chemicals present in the ground water and water bodies may lead to toxicity and pollution [154]. Hence, it is necessary to develop eco-friendly and biodegradable pesticides. In recent days, biopesticides are widely considered to be an alternative for synthetic pesticides; they are not harmful to humans or animals and are environmentally safe with added nutritive values to agricultural products [155]. Biopesticides’ synthesis through microbial fermentation is more costly than synthetic pesticides. However, some specific cyanobacteria are considered as effective biopesticides that can control the development of soil-borne diseases and fungal pathogens. In addition, biopesticides increase the defense systems of plants against pathogens [156]. Gupta et al. [157] reported that the isolation of active compounds called chlorellins from *Chlorella* sp. controls pathogen growth in plants. In another study, a compound, cryptophycin1 (depsipeptide) from the Nostoc sp. ATCC 53789, showed inhibitory activity against fungi and yeasts. In addition, cryptophycin 1 also effectively controlled Cryptococcus growth in plants by showing antiproliferative and antimitotic activities [158]. Latif et al. [159] reported that allelochemicals, called triketones, from microalgae control weed formation.

## 6. Wastewater Treatment by Microalgae

Recently, the interest in wastewater treatment-based microalgae biorefinery has attracted considerable attention due to the economical aspect of microalgae biorefinery implementation. Various wastewaters are rich in suitable nutrients, which can be utilized for microalgae production employing CO_2_ from atmosphere and flue gases. The major advantage of utilizing wastewater for microalgal production is that it not only addresses environmental issues but also produces renewable energy with high-value products [160]. A previous study successfully achieved microalgae (*Chlorella* sp.) production by utilizing municipal wastewater [142]. In addition, wastewater toxicity was efficiently reduced, including 70% of COD removal. Another study reported that 90% of wastewater nutrients including phosphorus and nitrogen was removed during wastewater purification utilizing microalgal cultivation [161]. A study reported that dairy wastewater can be efficiently utilized for the production of a mixture of microalgae such as *Chlorella* minutissima, Scenedesmus abundans, *Nostoc* muscorum, and *Spirulina* sp. [162]. In addition, energy consumption of conventional wastewater treatment methods is very high, up to 2 kW/h/m^3,^ whereas algal-based wastewater treatment technologies consume only 0.2 kW/h/m^3^. Similarly, energy consumption of microalgae-based wastewater treatment was reduced to 50% when compared to conventional wastewater treatment methods [163]. Hence, algae-based wastewater treatment is energy efficient for treating wastewater as well as producing microalgae rich in biofuel molecules. The representation of microalgal wastewater treatment and biofuel production is shown in Figure 2.

## 7. Technological and Economic Analysis

Microalgal biorefinery is the promising route to diminish atmospheric carbon dioxide and to utilize the poor-quality water and land to produce various valuable compounds including fuels. Recent studies suggested the utilization of microalgal feedstock in the progression of fuel conversion and other valuable products’ production. The development of various processing technologies and generation of productive pathways will allow for commercial feasibility in the future [164]. Cultivation of microalgae in a photobioreactor is an attractive option in terms of easy harvesting, high biomass productivity, less evaporation of medium water, easy control of the culture conditions, and low contamination risk [165]. However, microalgae cultivation in an open pond system is considered cheaper when compared to a photobioreactor [166]. The comparative analysis was done with 37.85 million L/year and the results showed that the total cost of algal oil production in an open pond system requires USD ($) 2.57/L whereas a photobioreactor requires USD ($) 5.45/L. Around 82% of total costs is required for the algal oil production in photobioreactors, which is three times higher than the total algal oil production cost with an open pond system. Hoffman et al. [167] assessed the techno-economic analysis of two different open pond cultivation systems, called open raceway pond and turf scrubber. The total cost required in terms of biomass production in an open raceway pond was USD 53.8/tonne, whereas the turf scrubber system required USD 704.4/tonne. The cost of biomass production in a photobioreactor was estimated at USD 3.903.4/kg dry biomass. However, substantial efforts in the aspect of engineering and technological advancements cut the biomass production cost and it could be as low as USD 0.57/kg dry biomass [168]. The maximum cost of biofuels produced from the microalgae was low, at USD 1.04/kg. Similarly, Wijffels et al. [169] proposed that the technological and engineering advancements could cut the cost to USD 0.50/kg biomass production; but, only sticking with the single-product biodiesel alone could not compete with the market value of other fuels. However, biorefinery approaches can fractionate other valuable compounds such as water-soluble proteins, pigments, and omega fatty acids, which improve the product value up to USD 34.38/kg. A 62% return on total investment can be retained within 2 years if the biorefinery approach is successfully designed [168]. The techno-economic assessment to produce biofuels and biochemicals by an integrated biorefinery with microalgae and Jatropha biomass showed that the total investment cost can be obtained within 3.3 years with the favorable production of biodiesel, glycerol by-product, de-oiled biomass for biogas, and spent biomass for animal feed. The coupling of biomass production with nutrient phytoremediation can save the cost by nearly USD 172.41/tonne of biomass [169]. Similarly, the overall biomass production cost can be retained by producing other valuable compounds. Other than biodiesel production from microalgal lipids, a part of lipids can be used for chemical coating and industrial applications (omega fatty acids), which can earn up to USD 2.47/kg. Another important component, protein, can be used as a food additive and animal feed, which gains USD 6.16/kg and USD 0.93/kg, respectively. The major source of microalgal biomass is carbohydrates, which can produce revenue up to USD 1.23/kg by utilizing carbohydrates for industrial purposes of bioenergy production [169]. Hence, the selection of microalgae is necessary for the accumulation of maximum target compounds due to the specific nature of microalgae. The strain improvement techniques or heterotrophic cultivation methods can improve the desired multiple production to benefit the profitable biorefinery. If strain improvement is successful with a high accumulation of high-value products such as β-carotene or astaxanthin, this can raise the market value of microalgal biomass to USD 100—1000/kg, which likely provides a profitable outcome [170]. Glycerol is the major by-product released during transesterification, which has a high industrial value and decreases the microalgal biodiesel production cost [170]. Purified glycerol obtained from the biodiesel production is successfully used for the fermentative H_2_ production from *R. Palustris*; purification of glycerol is necessary to avoid inhibition of the biomass growth during the fermentation process. [171]. Hence, the proper selection of microalgae for targeted compounds and the coupling of processing for various products’ production will generate a profitable microalgal biorefinery.

## 8. Commercial Microalgal Products Available in the Market

Spirulina is considered for the control of cholesterol and improvement of the immune system. Its sulfated polysaccharides are widely considered as antiviral agents. Since 1975, Japan has produced Spirulina tablets [172]. *Dunaliella* biomass was utilized for fish and animal feed [173]. The oxidized form of *Dunaliella* carotenoids is used as an anticancer agent. Antihypertensive, analgesic, and broncholytic drugs were made from *Dunaliella*. *Haematococcus* astaxanthin is used as a coloring agent in aquaculture to color fish muscles (salmon fish muscle) [174]. *Spirulina*, *Dunaliella,* and *Chlorella* have various health benefits, and the food industry makes tablets and capsules of these microalgae. They are also used along with noodles, biscuits, breads, candies, bean curd, and ice cream as a supplement. In addition, these microalgae are also used as a food additive to enhance nutritive health values [175,176]. D. salina was used in baking purposes, and *Chlorella* was used by beverage companies [176]. *Dunaliella* tablets are used as a vitamin A precursor due to its β-carotene property. Many antioxidant drugs can be produced from various microalgae. Phycobiliproteins are important components that could be commercialized soon. The omega fatty acid methyl ester could be used as a therapy for hypertriglyceridemia, and it could be commercialized soon [89]. The major microalgal products are biofuels and animal feed. However, primary products are not commercialized yet. To be more competitive, it is necessary to produce not only primary products such as biofuels but also to produce high-value products such as astaxanthin, β-carotene, EPA, DHA, natural dyes, bioactive compounds, antioxidants, and polysaccharides to enhance the economic feasibility of using microalgae as a feedstock.

## 9. Bottlenecks and Future Perspectives

The microalgal system is extensively considered for biofuel production. However, commercial microalgal production is still challenging due to the production cost. Therefore, regarding economic feasibility, other value-added products available from microalgae should be exploited. According to previous reports, other than biofuels, microalgae can produce high-value compounds such as pigments, microelements, omega fatty acids, antioxidants, animal feed, etc. However, downstream processing, such as the separation of multiple products and purification, is still challenging. To commercialize or scale up, some important factors need to be considered, for example, to design proper biomass production conditions to achieve a high biomass for targeted compounds, to eliminate the high-energy-requiring processes, and to introduce technologies in ultrafiltration and microfiltration for the availability of multiple products. The selection of a proper solvent system plays a vital role in biofuel production, which should be suitable and environmentally friendly. Microalgae can efficiently produce various products that can be consumed by humans; proper analysis is required to ensure the quality of products, which should comply with regulations.

Microalgae have the efficiency to produce biofuel and other value-added compounds in a biorefinery approach. However, different steps are involved in the production of various compounds with different purification approaches affecting the final product yield. Hence, to reduce the number of steps involved in the biorefinery approaches, it is necessary to couple the reactions to achieve higher final product yields. Some high-value products such as pigments are highly sensitive to light and temperature, which needs to be addressed. The separation of a protein–pigment complex is a challenging process and should be separated without affecting its functionality; preserving such proteins requires mild and non-invasive technologies [177]. Hence, the integration of both biofuel and other value-added products must be explored to lower the operating costs of microalgae biorefinery. In addition, research into the biology of cells and their metabolites should be investigated more extensively for value-added products. Intensive research is required to elucidate the biorefinery approach in environmental aspects by utilizing wastewater, which has a high nutrient content, to produce microalgal biomass and to fix CO_2_. On the other hand, industrial wastewater, with fewer nutrients and highly toxic compounds, is not suitable for algal production.

## 10. Conclusions

The application of a microalgal biorefinery approach has the huge potential to produce multiple products including biofuels. However, the possibility of producing multiple products economically requires more research. The coupling of various processes can reduce operation costs. Efficient biomass production is necessary to achieve a profitable microalgal biorefinery. Environmental factors including the utilization of wastewater and CO_2_ should be taken into consideration for successful biorefinery. The continuous production of biomass and its conversion to high-value products with efficient separation methods and low power consumption can lead to a successful operation of microalgal biorefinery in the near future.

## Figures and Tables

**Table 1:** Contents of macromolecules in various microalgae.

Microalgae Species	Carbohydrate (%)	Lipid (%)	Protein (%)	References
*Anabaena cylindrica*	25–30	4–7	43–56	[23]
*Arthrospira platensis*	15–25	4–7	55–70	[24]
*Chaetoceros calcitrans*	10	39	58	[25]
*Chaetoceros muellerii*	11–19	33	44–65	[25]
*Chaetoceros muelleri*	12–19	22–33	46–64	[26]
*Chlamydomonas rheinhardii*	17	21	48	[27]
*Chlorogloeopsis fritschii*	7	50	44	[28]
*Chlorella protothecoides*	10–15	55	10–52	[25]
*Chlorella vulgaris*	9–17	14–25	51–58	[27]
*Chlorella* *pyrenoidosa*	26	2	57	[27]
*Dunaliella salina*	32	6	57	[27]
*Dunaliella bioculata*	4	8	49	[27]
*Euglena gracilis*	14–20	14–18	39–61	[29]
*Euglena gracilis*	14–18	4–20	39–61	[25]
*Isochrysis galbana Parke*	7–25	21–38	30–45	[25]
*Nannochloropsis gaditana*	9.31	23.3	48.3	[30]
*Porphyridium cruentum*	40–57	9–14	28–39	[27]
*Porphyridium cruentum*	40–57	9–14	28–39	[23]
*Prymnesium parvum*	25–33	22–38	28–45	[27]
*Scenedesmus dimorphus*	18–52	16–43	8–18	[28]
*Scenedesmus quadricauda*	–	1.9	47	[27]
*Scenedesmus obliquus*	10–17	35–55	50–56	[23]
*Spirogyra* *sp.*	33–64	11–21	6–20	[27]
*Synechoccus sp.*	15	11	63	[27]
*Spirulina maxima*	13–16	6–7	60–71	[23]
*Spirulina platensis*	8–20	4–9	46–65	[25]
*Tetraselmis maculata*	15	3	52	[27]

**Table 2:** Ethanol production from various microalgae using different pretreatment and fermentative microorganisms.

Microalgal Strains	Pretreatment	Fermentative Microorganism	Fermentation Condition	Ethanol Production	References
*Chlamydomonas reinhardtii*	Enzymatic	*Saccharomyces cerevisiae S288C*	SSF, Temp: 30 °C, Time: 40 h, 160 rpm	0.235 (g/g algae)	[50]
*Chlorella*	Chemical (HCI and MgCI_2_)	*Saccharomyces cerevisiae Y01*	Temp: 30 °C, Time: 48 h, 200 rpm	22.60 (g/dm^3^)	[51]
*Chlorella* *variabilis*	Enzymatic	*Escherichiacoli KO11*	Temp: 35 °C, Time: 72 h, pH: 6.5, 150 rpm	0.326 (g/g carbohydrate consumed)	[52]
*Chlorella* *vulgaris*	Chemical (H_2_SO_4_)	*Escherichiacoli* *SJL2526*	SHF, Temp: 37 °C, pH: 7, 170 rpm	0.4 (g/g algae)	[53]
*Chlorella* *vulgaris FSP-E*	Chemical (H_2_SO_4_)	*Zymomonas mobilis ATCC 29191*	SHF, Temp: 30 °C, Time: 12 h, pH: 5–6	11.66 (g/dm^3^)	[54]
*Chlorococcum infusionum*	Chemical (NaOH)	*Saccharomyces cerevisiae*	Time: 12 h, 150 rpm	0.26 (g/g algae)	[55]
*Dunaliella tertiolecta*	Chemical HCl/H_2_SO_4_	*S. cerevisiae*	SHF, Temp: 30°C, Time: 12 h, 200 rpm	0.14 g/g algae	[56]
*Porphyridium cruemtum*	Enzymatic	*Saccharomyces cerevisiae KCTC 7906*	SSF, Temp: 37 °C Time: 9 h, pH: 4.8	2.77 (g/dm^3^) (seawater) 2.98 (g/dm^3^) (freshwater)	[57]
*Scenedesmus obliquus CNW-N*	Chemical (H_2_SO_4_)	*Zymomonas mobilis ATCC29191*	SHF, Temp: 30 °C Time: 4 h, pH: 6	8.55 (g/dm^3^)	Ho et al., 2013

**Table 3:** Microalgal bioproducts yields.

Microalgal Species	Products	Extraction and Purification Methods	Yield or Extraction Efficiency	Remarks	References
*Scenedesmus almeriensis*	Lipid	Solvent extraction [Soxhlet method: methanol–chloroform 2:1 (*v*/*v*)]	8.0 DW%	Need of organic solvent	[84]
*Scenedesmus almeriensis*	Lipid	ethanol:hexane (1:0.41 vol/vol)	19 DW%	Need of organic solvent	[118]
*Desmodesmus* sp.	Lipid	Chloroform:methanol (2:1 *v*/*v*)	5.6 g/L	Need of organic solvents	[119]
*Chlorella* sp.	Lipid	Isotonic extraction	19 wt.%	Energy intensive High capital cost	[17]
*Desmodesmus* sp.	Carbohydrates	Ultrsound + H_2_SO_4_ (10%)	5.2 g/L	Energy intensive and low extraction yield	[119]
*Chlorococcum infusionum*	Carbohydrates	Chemical hydrolysis (chemical pretreatment)	89.6% (sugar)	Relatively inexpensive	[93]
*Nannochloropsis salina*	Carbohydrates	H_2_SO_4_ (10%)	11.9 g/L	Cost effective	[120]
*Isochrysis aff. galbana*	Chlorophyll	Solvent extraction	5.6%	Organic solvent needed	[121]
*Haematococcus* *pluviali*	Astaxanthin	Solvent extraction	46 mg/L	Highest yield obtained with 6% CO_2_	[122]
*Chlorella saccharophila*	β-Carotene	Ultrasonication and cell disruption	37.3% (5.1 mg/g)	Improved extraction method	[107]
*Anabaena* sp. NCCU-9	Phycocyanin	Repeated freezing and thawing	128 mg/g	Optimization of culture conditions	[123]
*Chlorella saccharophila*	Zeaxanthin	Ultrasonication and cell disruption	72.2% (11.3 mg/g)	Improved extraction method	[107]
